# CBCT based online daily adaptive treatment improves target coverage with limited benefit for OAR protection in SBRT prostate cancer

**DOI:** 10.1186/s13014-026-02888-9

**Published:** 2026-07-07

**Authors:** Ádám Gáldi, Péter Ágoston, Csilla Pesznyák, Zoltán Takácsi-Nagy, Tibor Major

**Affiliations:** 1 Centre of Radiotherapy, National Institute of Oncology, Ráth György u. 7-9, Budapest, 1122 Hungary; 2 Institute of Nuclear Techniques, University of Technology and Economics, Budapest, Hungary; 3 Radiotherapy Department, Semmelweis University, National Institute of Oncology, Ráth György u.7-9, Budapest, 1122 Hungary; 4 Doctoral School, Semmelweis University, Budapest, Hungary; 5 National Tumor Biology Laboratory, National Institute of Oncology, Budapest, 1122 Hungary

**Keywords:** Online adaptive radiotherapy, SBRT, Prostate, IMRT, SIB, Dosimetry, CBCT, oART

## Abstract

**Background:**

The precise application of stereotactic body radiation therapy (SBRT) for prostate cancer is challenged by prostate motion and anatomical variations in surrounding organs such as the bladder and rectum. Online adaptive radiotherapy (oART) using cone beam computed tomography (CBCT) has been shown to improve target coverage while limiting doses to organs-at-risk (OARs).

**Methods:**

We retrospectively analyzed data from 30 prostate cancer patients who were treated with CBCT-based oART simultaneous integrated boost SBRT in five fractions, divided into two dose groups: lower dose group (LDG) receiving 30 Gy to seminal vesicles and prostate, and 35 Gy to prostate, and higher dose group (HDG) receiving 30 Gy to the vesicles and prostate, and 37.5 Gy boost to the prostate. Target volumes and OARs were contoured and evaluated per fraction. For each fraction, SCH (Scheduled plan) and ADP (Adaptive plan) plans were generated. Eight target volumes and fifteen OAR DVH (Dose Volume Histogram) parameters were collected and analyzed. Statistical comparisons were performed between differences in target coverages and doses to OARs in SCH and ADP plans, and between the LDG and HDG groups.

**Results:**

Target coverages were significantly improved in ADP plans in both cohorts and across the entire cohort (*p* < 0.001). SCH plans often failed to achieve the goal for PTV prostate. The median differences between target volume parameters of SCH and ADP plans were consistent, the adaptive plans were always superior regardless of the prescribed dose. Significant deterioration in ADP was found across the entire cohort in 7 OAR volumes. Dose reductions in OARs due to adaptation were less consistent. Significant differences were observed in bladder, rectum, sigmoid, femur, and penile bulb parameters in the sub-group analysis. The LDG-ADP plans resulted in slightly higher values of rectum_D0.04cm3_ and rectum_D50%_ than LDG-SCH, whereas in the HDG-ADP plans significantly lower doses for the rectum _D0.04cm3_, rectum_D1cm3_, bladder wall_V18.3 Gy_, bladder_D40%_ and higher value for bladder_V37Gy_, bladder wall_D15cm3_, penile bulb_D5 cm3_, penile bulb_D3cm3_, penile bulb_D5cm3_, sigmoid colon_D20cm3_ were achieved compared to the HDG-SCH plans. The magnitude of target coverage improvement was similar in the LDG and HDG groups, although higher prescribed doses were related to increased OAR doses in both SCH and ADP plans, particularly in the HDG group.

**Conclusions:**

CBCT-based online adaptive SBRT for prostate cancer significantly improves target coverage compared to the scheduled plans. Although the benefits of OARs dose vary, adaptive planning offers significant dosimetric advantages, especially at higher prescribed doses. These findings support the integration of oART into prostate SBRT to optimize the treatment’s efficacy and safety.

**Supplementary Information:**

The online version contains supplementary material available at https://doi.org/10.1186/s13014-026-02888-9.

## Introduction

In the modern era of radiotherapy, there are numerous options for the effective treatment of patients with early, organ-confined prostate cancer (PCa). This patient cohort could be treated with brachytherapy and external beam radiotherapy (EBRT) [1]. The stereotactic body radiation therapy (SBRT) is a commonly and widely used EBRT technique to treat low- and intermediate- risk PCa. Using this high-end technology is non-inferior, compared to the conventional radiotherapy approach in terms of tumour control and toxicity, confirmed in many clinical trials, for example in PACE B study, and in other prospective studies [2–6]. It is well known that the prostate gland and seminal vesicles are not independently moving organs, and their volume does not change rapidly. However, movements and forces caused by the surrounding organs, such as bladder, rectum, and intestines, can displace the prostate gland or the seminal vesicles, changing their shape [7–10]. Since we deliver high doses per fraction with high dose gradients and high conformity during SBRT treatments, high precision is required. Online adaptive radiotherapy (oART) is the technique that takes into account for daily positions of the organs and volume changes. It is proved, that oART is a well-applied radiotherapy approach to treat PCa patients [11–18]. In many cases we can achieve better dose coverage in terms of clinical target volume (CTV) and planning target volume (PTV), meanwhile the organs at risk doses could be lower than in non-adaptive technique. Waters et al. already confirmed that treating PCa patients with cone beam CT (CBCT) based online adaptive therapy, has a dosimetric benefit, based on data of 7 patients [13]. The clinical relevance of the improved dose coverage that can be achieved through adaptation may be a higher therapeutic dose delivered to the tumor-infiltrated organ, thereby presumably improving the tumor-killing effect, which may have an impact on local control and possibly on expected survival. In addition, to take into account the position of an OAR every day, we can achieve the same or better organ protection while increasing the target volume dose, so the number and severity of possible side effects are not expected to change. These results are mostly from dosimetric studies, so they will only really be clinically relevant when the long-term follow-up results come out. In our study, we retrospectively analysed 30 PCa patients who underwent oART with simultaneous integrated boost (SIB) SBRT treatments. We created two subgroups, based on the prescribed dose. Dose-volume parameters of organs at risk (OARs) and target volumes were evaluated on a fractional basis. Our goal was to investigate the benefit of oART SIB SBRT in PCa in a larger cohort of patients. In addition, we collected 23 different dosimetric parameters, and compared them in different dose cohorts. We hypothesized that we would find significant differences between scheduled and the daily online generated adapted radiotherapy plans, and prove the dosimetric benefit, in case of oART for SIB SBRT treatments.

## Methods

### Patient selection and groups

Thirty patients with low, intermediate and high risk PCa cancer (clinically N0M0) were selected for our evaluation. All of the patients were treated in 5 fractions with CBCT-based oART technique. The treated volumes were the prostate and the seminal vesicle (prostate and proximal seminal vesicle in one volume, called pvs), as weel the prostate itself (pros), treated simultaneously. Two groups, with fifteen patients in each, were created according to the prescribed dose. In the lower dose group (LDG), the seminal vesicles and prostate (prostate+seminal vesicles, pvs), were treated with 30 Gy, and simultaneously the prostate (prostate, pros) with 35 Gy. In the higher dose group (HDG), the seminal vesicles and prostate (pvs), were treated with 30 Gy, and simultaneously the prostate (pros) with 37.5 Gy. Based on our institutional protocol, patients aged between 70 and 75 years were treated with 37.5 Gy to the prostate, and patients older than 75 years with 35 Gy to the prostate. Note that in this study, high risk cases were also treated with local SBRT, since according to our institutional protocol, high-risk patients, localized PCa with clinical N0M0 stage above 70 years of age are not treated regionally, only locally to avoid toxicity. Therefore, none of the patients had received elective irradiation of lymph node region. Data from 30 patients were analysed, 25 of whom received hormone therapy. The average PSA level was 13.4 ng/ml (min. 0.15, max. 66.03), and the average Gleason score was 7 (min. 6, max. 9).

### Treatment planning and contouring

A Varian Ethos (Varian, Palo Alto, CA, USA) linear accelerator was used to treat the patients. This machine is suitable for performing CBCT-based oART treatments. The radiotherapy plans were generated in the dedicated Ethos treatment planning system V1.0. Intensity modulated radiotherapy (IMRT) plans with the sliding window technique were created. Plans made with 9 or 12 fields were selected depending on which plan resulted in the better reference plan. The linear accelerator is a 6 MV monoenergetic system, without a flattening filter. In the planning system, the minimum calculation grid size was 2.5 mm, and Acuros XB dose calculation algorithm was used. A template containing pre-defined clinical goals was used for all plans to create consistent plans. Beyond that, these goals helped to evaluate the adapted sessions. All of the plans were created with SIB technique. All reference plans and daily adapted plans were checked using Mobius 3D (Varian, Palo Alto, CA, USA) secondary dose calculation software, fraction by fraction.

CTV prostate volume (CTVpros) was contoured as the prostate. The CTVpvs volume was contoured as CTVpros plus the proximal seminal vesicles (1 cm of the vesicles was included in intermediate risk, and 2 cm of vesicles were included in high risk, non T3b cases, and all the vesicles were included in T3b cases where the tumour invaded the vesicles), and the prostate with a 5 mm margin, covering the microscopic spread, except towards the rectum where no margin was used. A 5 mm isotropic margin around the CTVs was used to create PTVpros and PTVpvs. Bladder, bladder wall, rectum, sigmoid colon, penile bulb, right and left femoral heads were contoured as OARs. These volumes are called influencer structures. The bladder wall was created with a 3 mm contour subtraction from the bladder contour. No rectal spacer was used.

In an oART session, the AI generated OAR volumes were evaluated and modified by a radiotherapy technologist and a physician. The original target volumes were deformed by the recontoured influencer structures, and these volumes were adjusted by a physician, fraction by fraction. There was a possibility to accept the reference plan, which is called the scheduled plan (SCH). In that case, the reference plan is re-calculated based on the daily anatomy using the original beam configurations and monitor units. The adapted plan (ADP) is a re-optimized plan that takes all anatomical changes into account.

The acceptance of the daily plan was performed by a physician who assessed the DVH, the traffic light system, and the isodose lines. The traffic light system is a decision support system, where the predefined goals are presented visually with green, yellow, or red colours indicating their fulfillment. The green means that the value is within tolerance, the yellow means it is within tolerance with minor deviation, and red means it is out of tolerance.

The goals were used uniformly for every patient and predefined as a template. Only three planning physicists were involved in the planning, and every plan underwent a multiple stages of review. This ensured the consistent quality of the plans. We also used standardized priorities, so the reference plans and the daily online adapted plans were consistent.

Our goals are listed in Table 1 of Supplementary Material.

### Dosimetric analysis

The treatment data were retrospectively analyzed. Parameters of eight different target volumes (TVs) and fifteen OARs were collected from five fractions of thirty patients.

The TV and OAR parameters are shown in Table 1 of Supplementary Material. We compared the SCH and ADP data per patient, also for the whole cohort, and separately for the LDG and HDG groups. We calculated the differences between SCH-ADP and HDG-LDG for each fraction and compared the medians of these values. We also calculated the median of the SCH, ADP, HDG, and LDG values.

### Statistical analysis

We used the Shapiro-Wilk test to determine the normality of the data distribution. To compare non-normally distributed data, we used the Wilcoxon signed-rank test for paired data, and the Mann-Whitney test for unpaired data. We applied Student’s t test for normally distributed data. We compared the SCH and ADP plans using paired tests, while the LDG and HDG groups were compared using unpaired tests. Bonferroni corrections were not applied. The difference was considered significant if the p-value was < 0.05. Prism 8.0.1 (GraphPad Software Inc., Boston, MA, USA) was used for statistical calculation.

## Results

All 30 patients were successfully treated in 5 fractions without any interruption, and modification of the fraction number. Out of 150 treatment sessions, SCH plan was chosen on only three occasions (2%). After consulting with the treating physician, he/she chose the SCH plan at his/her discretion because it provided better OAR protection. Evaluating the differences between target coverages in the whole cohort, an advantage was found with higher values in favour of the ADP plans (Table 1).

Considering all target volume parameters, a higher dose to the tumour was delivered in the ADP plan. For the PTVpros_V100%_ parameter, SCH plans failed to meet the minimum requirement of V100% > 90%.

**Table 1:** Median values with interquartile ranges of the dose-volume parameters of target volumes for SCH and ADP plans of all patients. The difference is the median of the fractional differences

	CTVprosV100%(%)	CTVpvs V100%(%)	PTVpros V100% (%)	PTVpros V95% (%)	PTVpros D0.01cm3 (%)	PTVpros Dmean (%)	PTVpvs V100% (%)	PTVpvs V95% (%)
SCH	99.4 (97.9–100.0)	100 (99.7–100.0)	89.9 (84.3–93.2)	97 (93.7 99.1)	113.0 (110.9-115.1)	104.8 (103.6-105.8)	95.6 (92.1–97.4)	97.6 (95.2–98.8)
ADP	100 (99.8–100.0)	100 (100.0-100.0)	95.1 (92.2–97.3)	99.4 (97.4–99.9)	114.0 (112.4-116.5)	106.2 (105.0-107.3)	99.1 (98.0-99.6)	100 (99.7–100.0)
Difference (ADP-SCH)	-0.4 (-1.7-0.0)	0.0 (-0.3-0.0)	-5.4 (-10.0-( -1.2))	-1.7 (-4.2-( -0.1))	-1.0 (-3.5-0.7)	-1.6 (-3.0-(-0.3))	-3.3 (-6.3-(-1.0))	-2.2 (-4.7-( -0.8))
P value	**< 0.001**	**< 0.001**	**< 0.001**	**< 0.001**	**< 0.001**	**< 0.001**	**< 0.001**	**< 0.001**

SCH: scheduled plan in which the dose is re-calculated on daily anatomy without adaptation, ADP: adapted plan in which the dose is re-optimised on daily anatomy

**Fig. 1:**
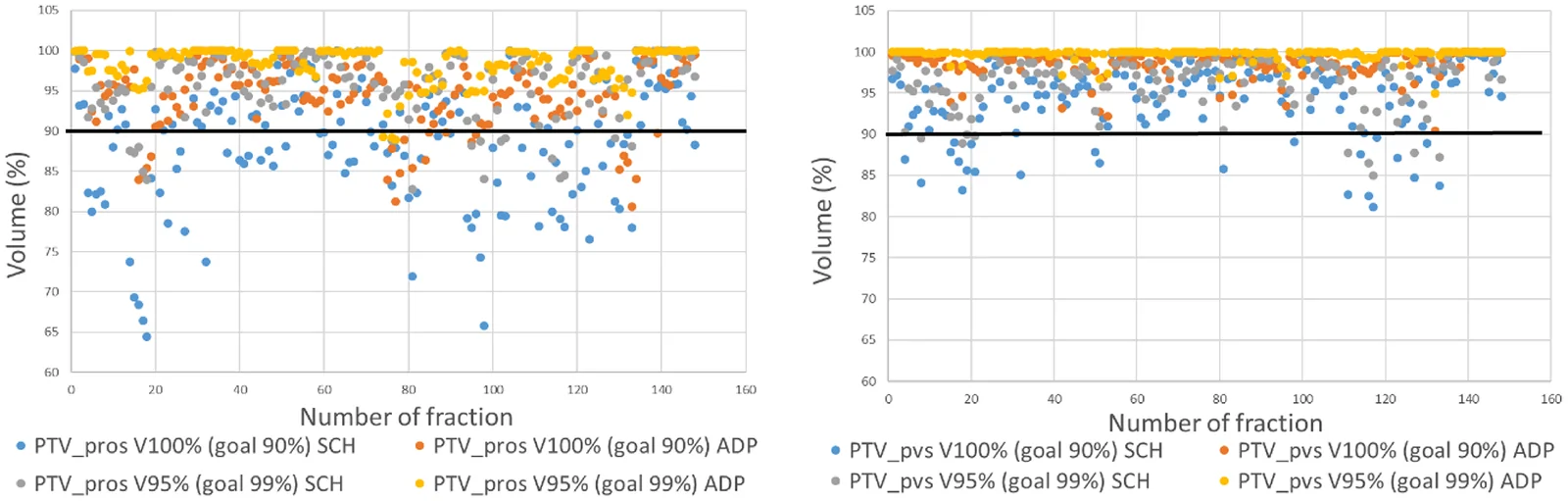
Target volume coverages of the PTV_pros_ (left), and PTV_pvs_ (right) in the function of fractions. The black line shows the goal of V100%

Figure 1 shows that the parameters analysed for the prostate have a much larger variance compared to the parameters for the PVS when analyzed over the whole cohort in every fraction. The left figure shows the percentage volumes of four different PTVpros parameters in every fraction, and the right figure shows the volumes of four different PTVpvs parameters in every fraction. In both volumes, deviations between the ADP plans are smaller than those between the SCH plans.

In the cases of the bladder wall_D15cm3_, rectum_D50%_, bladder_D40%_, penile bulb_D5cm3_, penile bulb_D3cm3_, sigmoid colon_D0.04cm3_, and sigmoid colon_D20cm3_, significantly higher values were observed in the ADP plans, and lower for sigmoid colon_D0.04cm3,_ and Bladder wall_V18.3 Gy_ than in the SCH plans (Table 2).

**Table 2:** Median values with interquartile range of the dose-volume parameters for OARs in the SCH and ADP plans in all patients. The difference is the median of the fractional differences

	Bladder wall V18.3 Gy (cm3)	Bladder wall D15cm3 (Gy)	Bladder wall D0.04cm3 (Gy)	Rectum D1cm3 (Gy)	Rectum D0.04cm3 (Gy)	Rectum D50% (Gy)	Rectum D20% (Gy)	Bladder D40% (Gy)
SCH	15.6 (11.9–19.7)	2.7 (2.0-4.4)	7.7 (7.4–8.2)	6.9 (6.7–7.2)	7.3 (7.1–7.6)	3.1 (2.9–3.4)	4.6 (4.2-5.0)	2.3 (1.5–3.2)
ADP	14.4 (12.4–16.6)	2.9 (1.9–3.9)	7.8 (7.4–8.2)	6.9 (6.6–7.1)	7.3 (7.1–7.5)	3.2 (3.0-3.5)	4.7 (4.3-5.0)	2.3 (1.6-3.0)
difference (ADP-SCH)	-0.3 (-0.8-2.7)	0.0 (0.0-0.3)	0.0 (-0.2-0.1)	0.0 (-0.1-0.2)	0.0 (-0.2-0.3)	0.1 (-0.3-0.1)	0.0 (-0.3-0.2)	0.1 (-0.1-0.4)
P value	**0.032**	**0.025**	0.238	0.107	0.209	**0.002**	0.320	**0.005**

	Bladder _V37Gy_ (cm^3^)	Penile bulb _D5cm3_ (Gy)	Penile bulb _D3cm3_ (Gy)	Sigmoid colon _D0.04cm3_ (Gy)	Sigmoid colon _D20cm3_ (Gy)	Femur h. left _D10cm3_ (Gy)	Femur h. right _D10cm3_ (Gy)	
SCH	1.6 (0.1–4.5)	0.8 (0.5–2.4)	0.5 (0.4–0.8)	0.5 (0.3–1.5)	0.2 (0.2–0.4)	2.7 (2.3–3.1)	2.7 (2.4-3.0)	
ADP	1.8 (0.0-5.4)	1.2 (0.6–2.3)	0.6 (0.4–0.8)	0.5 (0.4–2.1)	0.2 (0.2–0.4)	2.7 (2.3–3.1)	2.6 (2.3-3.0)	
difference (ADP-SCH)	0.0 (-0.4-1.4)	0.1 (-0.6-0.1)	0.0 (-0.1-0.0)	0.0 (-0.1-0.0)	0.0 (0.0–0.0)	0.0 (-0.1-0.2)	-0.1 (-0.1-0.2)	
P value	0.185	**0.005**	**0.011**	**0.007**	**0.025**	0.203	**< 0.001**	

SCH: scheduled plan in which the dose is re-calculated on daily anatomy without adaptation, ADP: adapted plan in which the dose is re-optimised on daily anatomy

We also performed analyses on paired SCH and ADP plans. The differences between the two groups are presented in Table 3. The median values of parameters for target volumes in SCH and ADP plans were grouped by the prescribed dose. We found that all ADP plans showed better results by the same magnitude compared to SCH, regardless of the prescribed dose (ADP-SCH difference). It can be seen from the data that all of the prostate target volume parameters show significantly lower values in the HDG group, compared to LDG, except for the D0.01cm^3^ parameter, which was higher.

**Table 3:** Median values with interquartile ranges of dose-volume parameters for target volumes in patients in the higher dose group (HDG) and lower dose group (LDG) with the corresponding SCH and ADP dose parameters. The differences are calculated from medians of the fractional differences for HDG and LDG, as well as ADP and SCH groups

	Type of plan	HDG	LDG	difference (HDG-LDG)	*P* value
CTVpros_V100%_ (%)	SCH	98.6 (97.3–99.6)	99.9 (99.0-100)	-0.5 (-2.1-0.1)	< 0.001
	ADP	99.8 (99.6–99.9)	100.0 (100–100)	-0.2 (-0.4-0.0)	**< 0.001**
difference (ADP-SCH)		1.0 (0.0-2.7)	0.1 (0.0–1.0)		
P value		**< 0.001**	**< 0.001**		
CTVpvs_V100%_ (%)	SCH	100 (99.7–100)	100 (99.6–100)	0.0 (-0.2-0.3)	0.523
	ADP	100 (100–100)	100 (100–100)	0.0 (0.0–0.0)	0.242
difference (ADP-SCH)		0.0 (0.0-0.3)	0.0 (0.0-0.4)		
P value		**< 0.001**	**< 0.001**		
PTVpros_V100%_ (%)	SCH	87.9 (82.3–91.9)	91.1 (87.0-94.3)	-2.2 (-9.5 2.1)	**0.005**
	ADP	93.9 (90.9–95.4)	97.1 (94.3–98.6)	-3.2 (-6.2-(-1.2))	**< 0.001**
difference (ADP-SCH)		5.5 (-0.1-9.1)	5.3 (1.5–10.1)		
P value		**< 0.001**	**< 0.001**		
PTVpros_V95%_ (%)	SCH	95.1 (91.7–97.6)	98.5 (96.9–99.4)	-2.7 (-6.4-0.6)	**< 0.001**
	ADP	97.5 (95.9–98.6)	99.9 (99.7–100)	-2.4 (-3.5-(-0.4))	**< 0.001**
difference (ADP-SCH)		2.5 (-0.1-4.7)	1.4 (0.3–3.1)		
P value		**< 0.001**	**< 0.001**		
PTVpros_D0.01cm3_ (%)	SCH	114.0 (112.8-115.1)	111.6 (110.3-114.7)	1.9 (-1.9-4.6)	**0.003**
	ADP	114.8 (113.2-116.7)	113.2 (111.4–116)	1.4 (-0.5-3.8)	**0.003**
difference (ADP-SCH)		1.0 (-0.7-3.1)	1.1 (-0.9-3.7)		
P value		**< 0.001**	**< 0.001**		
PTVpros_Dmean_ (%)	SCH	105.1 (103.5-106.5)	104.7 (103.8-105.4)	0.4 (-0.8-1.6)	0.200
	ADP	106.5 (105.4-107.5)	105.9 (104.8-107.2)	0.4 (-1.1-2.1)	0.196
difference (ADP-SCH)		1.6 (0.6–2.5)	1.6 (0-3.4)		
P value		**< 0.001**	**< 0.001**		
PTVpvs_V100%_ (%)	SCH	95.6 (92.4–97.5)	95.7 (92.0-97.3)	0.6 (-4.3-4.5)	0.863
	ADP	99.2 (98.0-99.5)	98.8 (97.9–99.8)	-0.2 (-1.1-1.3)	0.704
difference (ADP-SCH)		3.3 (1.0-6.8)	3.3 (1.3–6.1)		
P value		**< 0.001**	**< 0.001**		
PTVpvs_V95%_ (%)	SCH	97.4 (94.4–98.7)	97.8 (95.7–98.9)	-0.3 (-3.1-2.4)	0.306
	ADP	99.9 (99.6–100)	99.9 (99.8–100)	0.0 (-0.4-0.1)	0.331
difference (ADP-SCH)		1.9 (0.6–4.8)	2.3 (1.0-4.1)		
P value		**< 0.001**	**< 0.001**		

SCH: scheduled plan in which the dose is re-calculated on daily anatomy without adaptation, ADP: adapted plan in which the dose is re-optimised on daily anatomy

For 14 OAR parameters, we found that the HDG had significantly higher dose parameters in both SCH and ADP plans (Table 4). The rectum_D1cm3_ parameter was an exception, because the ADP value was lower in the HDG group (see Table 4, difference (HDG-LDG); -0.22). A relatively large difference was found between the LDG and HDG groups for the bladder_V37Gy_ parameter. This difference was observed in SCH and in ADP plans, too. In the case of the HDG, even the ADP plans slightly exceeded the limit of our protocol.

**Table 4:** Median values with interquartile ranges of dose-volume parameters for target volumes in patients in the higher dose group (HDG) and lower dose group (LDG) with the corresponding SCH and ADP dose parameters. The differences are calculated from medians of the fractional differences for HDG and LDG, as well as ADP and SCH groups

	Type of plan	HDG	LDG	difference (HDG-LDG)	*P* value
Bladder wall_V18.3 Gy_ (cm^3^)	SCH	14.5 (12.2–17.5)	17.5 (14.1–20.3)	-4.0 (-7.5-4.8)	0.613
	ADP	13.5 (11.2–15.1)	17.3 (13.6–19.6)	-4.0 (-8.3-2.3)	0.639
difference (ADP-SCH)		-0.7 (-2.0-1.6)	0.1 (-0.6-1.1)		
P value		**0.004**	0.573		
Bladder wall_D15cm3_ (Gy)	SCH	2.5 (1.9–4.8)	2.8 (2.0-4.1)	0.2 (-1.8-1.7)	0.436
	ADP	2.8 (1.8–4.5)	2.9 (2.0-3.7)	0.1 (-1.1-1.3)	0.591
difference (ADP-SCH)		-0.1 (-0.4-0.1)	-0.1 (-0.5-0.2)		
P value		**0.010**	0.456		
Bladder wall_D0.04cm3_(Gy)	SCH	8.2 (7.9–8.4)	7.4 (7.3–7.5)	0.8 (0.6-1.0)	**< 0.001**
	ADP	8.2 (7.9–8.4)	7.4 (7.3–7.5)	0.7 (0.4–0.9)	**< 0.001**
difference (ADP-SCH)		0.0 (-0.2-0.1)	0.1 (-0.1-0.2)		
P value		0.495	**0.008**		
Rectum_D1cm3_ (Gy)	SCH	7.0 (6.6–7.3)	6.9 (6.7-7.0)	0.0 (-0.4-0.4)	0.338
	ADP	6.7 (6.3–7.1)	7.0 (6.8–7.1)	-0.3 (-0.5-0.1)	**< 0.001**
difference (ADP-SCH)		-0.1 (-0.5-0.0)	0.00 (-0.1-0.2)		
P value		**0.001**	0.116		
Rectum_D0.04cm3_ (Gy)	SCH	7.6 (7.3–7.8)	7.2 (7.0-7.4)	0.3 (-0.1-0.7)	0.899
	ADP	7.4 (7.2–7.5)	7.3 (7.1–7.5)	0.0 (-0.2-0.3)	0.493
difference (ADP-SCH)		-0.2 (-0.4-0.0)	0.1 (-0.1-0.2)		
P value		**0.003**	**0.008**		
Rectum_D50%_ (Gy)	SCH	3.2 (3.0-3.7)	3.0 (2.8–3.2)	0.2 (-0.1-0.7)	**< 0.001**
	ADP	3.3 (3.0-3.7)	3.1 (3.0-3.3)	0.1 (-0.1-0.5)	**0.018**
difference (ADP-SCH)		0.0 (-0.2-0.3)	0.1 (-0.1-0.3)		
P value		0.329	**< 0.001**		
Rectum_D20%_ (Gy)	SCH	4.7 (4.4–5.2)	4.5 (4.2–4.9)	0.3 (-0.5-0.8)	**0.023**
	ADP	4.7 (4.3–5.2)	4.7 (4.3–4.9)	0.1 (-0.3-0.4)	0.247
difference (ADP-SCH)		0.0 (-0.3-0.3)	0.0 (-0.1-0.3)		
P value		0.784	0.054		
Bladder_D40%_ (Gy)	SCH	2.8 (1.8–3.5)	2.1 (1.3–2.7)	0.8 (-0.7-1.8)	**< 0.001**
	ADP	2.4 (1.8–3.2)	2.1 (1.5–2.7)	0.5 (-0.5-1.1)	**0.023**
difference (ADP-SCH)		-0.3 (-0.7-0.0)	0.0 (-0.2-0.2)		
P value		**< 0.001**	0.358		
Bladder_V37 Gy_ (cm^3^)	SCH	4.6 (2.8–11.3)	0.1 (0.0-0.4)	4.3 (2.4–9.3)	**< 0.001**
	ADP	5.3 (2.9–6.9)	0.1 (0.0-0.4)	4.9 (2.3–6.5)	**< 0.001**
difference (ADP-SCH)		-1.3 (-3.4-1.0)	0.0 (-0.1-0.2)		
P value		**0.007**	0.092		
Penile bulb_D5cm3_ (Gy)	SCH	0.8 (0.5–2.3)	0.7 (0.5–2.7)	0.0 (-0.8-0.5)	0.244
	ADP	1.3 (0.7–2.6)	0.9 (0.5–2.1)	-0.1 (-0.5-0.9)	0.080
difference (ADP-SCH)		0.0 (0.0-0.5)	0.0 (-0.2-0.5)		
P value		**0.004**	0.211		
Penile bulb_D3cm3_ (Gy)	SCH	0.5 (0.4–0.7)	0.4 (0.4-1.0)	0.0 (-0.4-0.2)	0.068
	ADP	0.6 (0.5–0.8)	0.5 (0.4–0.8)	0.1 (-0.3-0.3)	**0.007**
difference (ADP-SCH)		0.0 (0.0-0.2)	0.0 (-0.1-0.1)		
P value		**0.014**	0.240		
Sigmoid colon_D0.04cm3_ (Gy)	SCH	0.7 (0.4–2.9)	0.4 (0.3–0.7)	0.0 (-0.4-1.2)	**0.015**
	ADP	0.7 (0.4–3.2)	0.4 (0.3–0.9)	0.0 (-0.4-1.8)	**0.008**
difference (ADP-SCH)		0.0 (0.0-0.1)	0.0 (0.0-0.1)		
P value		0.057	0.059		
Sigmoid colon_D20cm3_ (Gy)	SCH	0.3 (0.2–0.4)	0.2 (0.2–0.3)	0.1 (-0.1-0.2)	**0.004**
	ADP	0.3 (0.2–0.4)	0.2 (0.1–0.3)	0.0 (-0.1-0.2)	**0.007**
difference (ADP-SCH)		0.0 (0.0–0.0)	0.0 (0.0–0.0)		
P value		**0.035**	0.347		
Femur h. left_D10cm3_ (Gy)	SCH	2.8 (2.3–3.3)	2.7 (2.3–2.9)	-0.2 (-0.6-0.9)	0.271
	ADP	2.8 (2.4–3.1)	2.6 (2.3–2.9)	0.1 (-0.4-0.8)	0.097
difference (ADP-SCH)		0.0 (-0.1-0.2)	-0.1 (-0.2-0.1)		
P value		0.762	**0.021**		
Femur h. right_D10cm3_ (Gy)	SCH	2.8 (2.4–3.2)	2.7 (2.5–2.9)	0.0 (-0.5-0.5)	0.402
	ADP	2.7 (2.3–3.2)	2.6 (2.3–2.9)	0.1 (-0.5-0.7)	0.291
difference (ADP-SCH)		0.0 (-0.2-0.1)	-0.1 (-0.2-0.0)		
P value		0.084	**< 0.001**		

SCH: scheduled plan in which the dose is re-calculated on daily anatomy without adaptation, ADP: adapted plan in which the dose is re-optimised on daily anatomy

In the case where the two groups were analysed separately, we found that in all cases the ADP plans achieved significantly better target coverage than the SCH plans (Table 3). In terms of OARs, in the LDG group, the bladder wall_D0.04cm3_, rectum_D0.04cm3_, rectum_D50%_, parameters were significantly higher, and the left and right femur h._D10 cm3_ were lower in the ADP plans than in the SCH plans (Table 4).

In the HDG group, the value of the bladder wall_V18.3 Gy_, rectum_D1cm3_, rectum_D0.04cm3_, bladder_D40%_ were significantly lower, but bladder wall_D15cm3_, bladder_V37Gy_, penile bulb_D5 cm3_, penile bulb_D3cm3_, and sigmoid colon_D20cm3_ were higher.

**Fig. 2:**
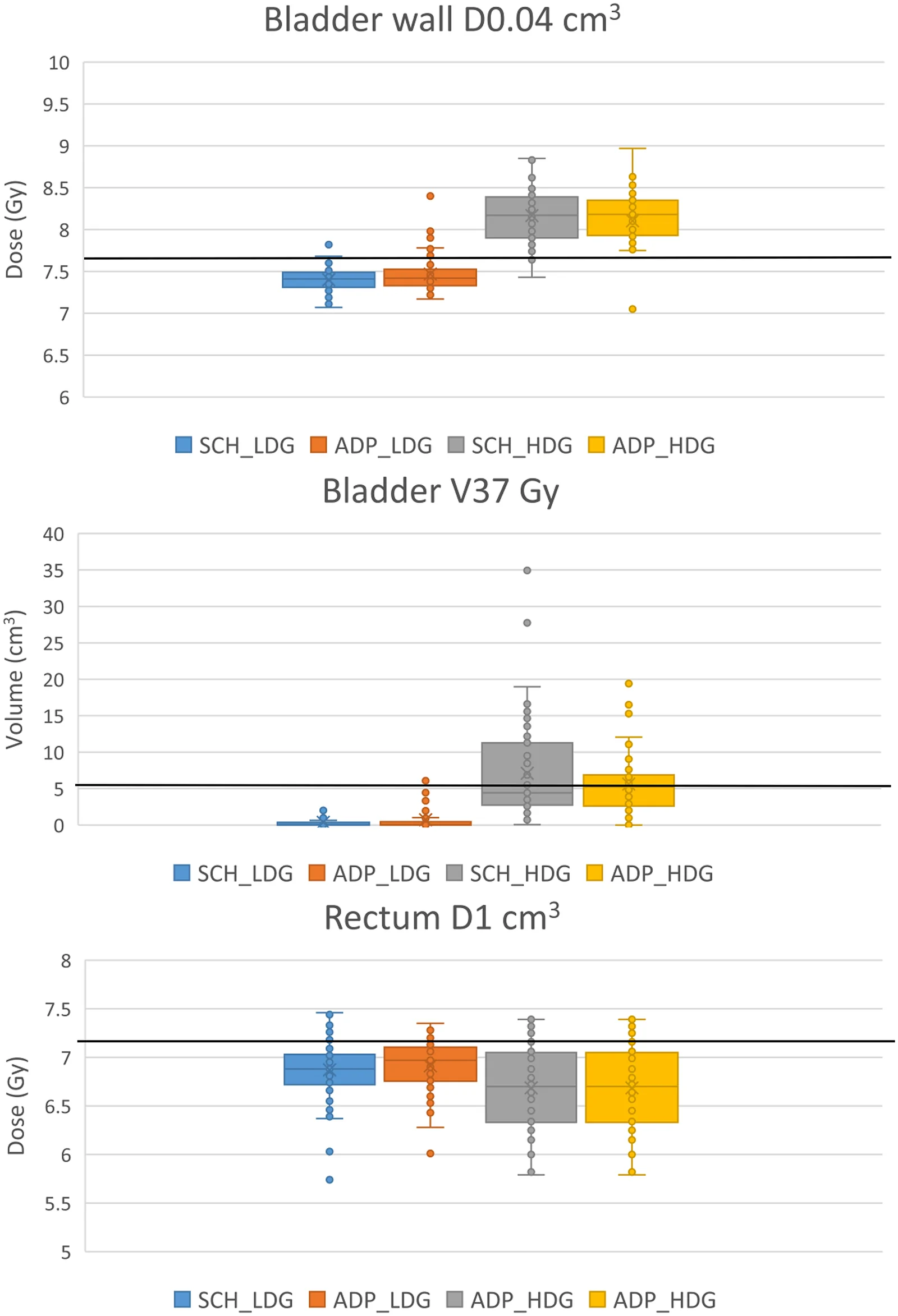
Box and whiskers plot of the dose-volume parameters of OARs where significant differences were observed. The boxes represent the medians and interquartile ranges (75th and 25th percentiles). SCH: scheduled, ADP: adaptive, LDG: lower dose group, HDG: higher dose group. The black lines show the institutional OAR goals

In Fig. 2, we show 3 different evaluated parameters, where a significant difference was found between HDG and LDG.

## Discussion

In this work, using a large number of patients and fractions, we demonstrated that online adaptation can be beneficial for patients with prostate tumour undergoing SBRT radiotherapy. In our study, we found a clear and significant advantage of adaptation in all eight TV parameters for the entire cohort. The V100% parameter of the PTVpros must be at least 90%, but in the SCH plans, we found a median value of 89.9%, which is less than required in our institutional and international protocols [19]. However, in the ADP plans, in all cases we were able to meet the acceptance criterion, similar to Waters et al. [13]. Fisher and colleagues also achieved similarly good target volume coverage, but in their study, they analyzed data from only 6 patients, and evaluated the D98% values, whereas we used the V100% and V95% in our study. However, they found differences in D98% of PTV with significant improvement with adaptation (*p* < 0.0001; median D98% 91.8–96.5% [14]. With regard to the OARs, we found that, for 7 of the parameters examined, the ADP group had significantly higher values, and for two parameters, lower than the SCH group, when the entire cohort was analyzed. We found this result surprising because it did not support our hypothesis. It should be noted that the differences were minimal. Compared with the findings of Fisher and colleagues, no clear advantage was demonstrated in terms of organ protection either, rectum_V40Gy_ median dose values were 24% vs. 19.8% in SCH vs. ADP, bladder V40Gy median dose values were 31% vs. 32.6% in SCH vs. ADP (both *p* < 0.001). It should be noted that fewer parameters of organs were evaluated (V40Gy and mean dose for rectum, V40Gy and mean dose for bladder, D0.1cm^3^ for bowel), and the power of significance was reduced due to the low number of patients [14]. In contrast, Waters et al. [13] found that there was a small but statistically significant difference in the rectum D0.03cm^3^ parameter for ADP plans, and a minimal dose increase for the bladder parameter when the ADP plans were considered for the whole cohort.

We observed that, regardless of the prescribed dose, all ADP plans showed a similar improvement in prostate target volume parameters compared to the SCH plans. This is possible because we used similar clinical goals for optimization. We observed that the target volume parameters for prostate were significantly lower in SCH and ADP plans in the HDG group compared to the LDG group. This can be explained by the fact that we had to reduce the dose coverage of the target volumes in the HDG group if the OAR dose limitations were prioritized (but still within the target acceptance criteria) to keep the dose to surrounding critical organs within predetermined dose limits. In favour of ADP, the largest deviation was found in PTVpros_V100%_ (HDG: 93.9 (90.9–95.4) and LDG: 97.1 (94.3–98.6)). The PTVpros_D0.01cm3_ was an exception, it was higher in the HDG group. This is because, with higher dose treatment, in order to achieve the same OARs protection as for lower dose treatment, the TPS has to create a higher dose maximum to achieve a larger dose fall off.

When subgroups with lower and higher prescribed doses were analysed separately, there was no clear improvement in favour of the ADP in terms of OARs. In the LDG, the bladder wall_D0.04cm3_, rectum_D0.04 cm3_, rectum_D50%_, parameters were significantly higher, and the left and right femur h._D10cm3_ were lower in ADP plans than in SCH plans. In reviewing these plans, we found that, based on the clinical goals used in the reference plan, the system prioritized target volume coverage over the OAR protection during the re-optimization of the ADP. Thus, coverage of the target volume was of paramount importance, but at the same time, the acceptance criteria for the organs at risk in the ADP also had to be met. In contrast, in the HDG group, significantly lower dose values were obtained in ADP plans for the bladder wall_V18.3 Gy_, rectum_D1cm3_, rectum_D0.04cm3_, bladder_D40%_, but for the bladder wall_D15cm3_, bladder_V37Gy_, penile bulb_D5cm3_, penile bulb_D3cm3_, sigmoid colon_D20cm3_ the doses were higher than in the SCH plans. Because the decimal places had to be rounded, the slight differences between the values may not always be clearly visible in the tables, in some cases. In this group, the prescribed dose was 7.5 Gy per fraction. This dose level has proven to be high enough in nearly all treatments to allow for a much greater emphasis on protecting the OARs. Consequently, the optimization criterion for the rectum and bladder in the reference plans, and thus also in the re-optimized plans, resulted in lower doses for the two organs to be protected. With regards to the bladder wall_D15cm3_ parameter, we found that this parameter is generally an easily achievable clinical goal, in contrast to the requirements for the maximum dose for the rectum, so we allowed a higher dose at the expense of the bladder wall_D15cm3_ parameter, to meet the maximum requirements. For similar reasons, we allowed higher doses for adaptive plans with other organs. In terms of bladder_V37Gy_, we found that the larger volume of the bladder receives a higher dose during adaptation, but this may be because in the HDG group a larger proportion of the vesicles had to be included in the target volume, so that, taking the daily anatomical movement into account this was essential for adequate coverage of the vesicles. In summary, by prioritizing the goals of the targets, the planning system is able to maintain the dose to organs at risk even when there is potential overlap between the target volume and organs at risk.

When comparing data for OARs in the LDG and HDG groups, we found significantly lower median dose values in the LDG group in both the SCH and ADP plans for 14 parameters. These results can be explained by the fact that the prescribed dose was lower in the LDG group. In the HDG group, we found that the rectum_D1cm3_ parameter was significantly lower in the ADP plans, than in the LDG group. This can be explained by the fact that the rectum is a critical organ, and its wall is in close contact with the prostate. This makes it very difficult to meet the dose limits when a higher prescribed dose is applied. Therefore, when planning, we must prioritize the protection of this organ, even at the expense of the prostate.

When high-dose online adaptive prostate treatments were implemented, we expected that the maximum dose to the rectum would decrease in all cases with adaptive plans, regardless of the prescribed doses. However, the analyzed data did not support our initial hypothesis, on the other hand, theydemonstrated that this technique can be safely used with regard to rectal dose. Based on the entire cohort, we observed more significant deterioration in the ADP group; however, at sub-group analysis, we were able to achieve a significantly lower dose for maximal rectum dose in the HDG group. Furthermore, it has been confirmed that this technique can be safely used with regard to the rectal dose.

There are several scientific papers in the literature that have demonstrated the combination of target volume advantage and OAR protection in oART [14, 20–24]. However, in most cases, the number of patients was low, but the fraction number was high. The question is whether we can apply our results to larger patient population. It is worth mentioning the work of Prezios et al. [25], who performed dosimetric analysis on prostate cancer patients treated with conventional dose fractionation with CBCT-based adaptive treatment. Their results were similar to ours, with clear advantages in terms of OAR protection.

To the best of our knowledge, this is the largest study to examine the dosimetric efficacy of the CBCT-based online adaptive stereotactic therapy in a cohort of prostate cancer patients, and the only study to examine such a large number of dosimetric parameters of target volumes and organs at risk, comparing two different prescribed dose groups.

After evaluating the very early and early side effects of our first treated 30 patients, the next step for our group will be to investigate how we can decrease the rate of side effects or increase the local control with oART in prostate cancer compared to other external beam radiotherapy techniques. However, based on the excellent results of phase 3 SBRT studies in the international literature [26, 27], we do not expect large differences in toxicity in OARs. Rather, we expect that slightly better local control can be achieved through improved dose coverage of the target volumes.

It is evident that we have excellent coverage of CTVs in all cases, but we must not forget that the adaptation time is long (approx. 15 min), and the treatment time is approx. 4 min. We take a verification image before starting the irradiation, but residual errors may still remain, not to mention anatomical changes during the treatment. We therefore believe that, given the current situation the examination of dose to the PTV cannot be completely disregarded. However, in light of the excellent PTV coverage observed in this study, it is reasonable to consider reducing the CTV-PTV margin. The rationale for reducing OAR doses will be confirmed or refuted when the first clinical results become available with a comparison of toxicities across a large numbers of patients.

We also plan to analyse variations in fraction doses on an individual patient basis and conducti research on the possibility of reducing the PTV margin. This work is ongoing, and the manuscript is under preparation.

## Conclusions

In summary, we have successfully introduced an online adaptive stereotactic simultaneous integrated boost technique with online adaptive treatment for prostate cancer patients at our institute. After analyzing the treatment data of 30 patients, we found that the target coverage parameters had improved significantly. In one of the evaluated volumes, only the daily adapted treatment was clinically acceptable.

In contrast, an analysis of the entire patient population revealed no clear benefit of adaptation with regard to the organs at risk However, this finding shows that the target volume coverage can be improved while keeping the dose to OARs at acceptable level, regardless of the prescribed dose. In a subgroup analysis, we found significant differences in several organs at risk. In most cases, the difference occurred when the prescribed dose was higher, and the treatment planning system had to work harder to meet the dose requirements.

### Limitation

A limitation of our study is that the daily adapted contouring was not always performed by the same physician, so the results may be influenced by intraobserver variability. In addition, there was also slight difference between the two dose groups in the contouring protocol regarding the target volumes, depending on the extent of the tumour, which may also have had a minimal effect on the dosimetric results. We cannot ignore the fact that further anatomical changes may occur during the irradiation after the adaptation phase [17, 23, 28]. The average irradiation time is approximately 4 min, during which time online verification is not possible.

## Supplementary Information

Below is the link to the electronic supplementary material.

Supplementary Material 1

## Data Availability

Measurement data can be disclosed on request.

## Abbreviations

ADP: Daily adapted plan
AI: Artificial intelligence
CBCT: Cone Beam Computed Tomography
CT: Computer tomography
CTV: Clinical target volume
GTV: Gross tumor volume
HDG: Higher dose group
IMRT: Intensity-modulated radiotherapy
LDG: Lower dose group
LINAC: Linear Accelerator
OAR: Organ-at-risk
oART: Online adaptive radiotherapy
PCa: Prostate cancer
PTV: Planning target volume
PVS: Prostata plus vesicles seminal
SBRT: Stereotactic body radiation therapy
SCH: Scheduled plan
SIB: Simultaneous integrated boost
TPS: Treatment planning system

## References

[1] Sekhoacha M, Riet K, Motloung P, Gumenku L, Adegoke A, Mashele S. Prostate cancer review: genetics, diagnosis, treatment options, and alternative approaches. Molecules. 2022 Sep 5;27(17):5730. 10.3390/MOLECULES27175730. PMC9457814 36080493

[2] van As N, et al. 5-Year outcomes from PACE B: an international phase iii randomized controlled trial comparing stereotactic body radiotherapy (SBRT) vs. conventionally fractionated or moderately hypo fractionated external beam radiotherapy for localized prostate cancer. Int J Radiat Oncol. Nov. 2023;117(4):e2–3. 10.1016/j.ijrobp.2023.08.027.

[3] Jackson WC, Silva J, Hartman HE, Dess RT, Kishan AU, Beeler WH, et al. Stereotactic body radiation therapy for localized prostate cancer: a systematic review and meta-analysis of over 6,000 patients treated on prospective studies. Int J Radiat Oncol Biol Phys. 2019 Jul 15;104(4):778-789. 10.1016/J.IJROBP.2019.03.051. PMC6770993 30959121

[4] Anwar M, Weinberg V, Chang AJ, Hsu IC, Roach M, Gottschalk A. Hypofractionated SBRT versus conventionally fractionated EBRT for prostate cancer: Comparison of PSA slope and nadir. Radiat Oncol. Feb. 2014;9(1):1–9. 10.1186/1748-717X-9-42/TABLES/6. PMC3923240 24484652

[5] Chen LN, et al. Stereotactic body radiation therapy (SBRT) for clinically localized prostate cancer: The Georgetown University experience. Radiat Oncol. Mar. 2013;8(1):1–10. 10.1186/1748-717X-8-58/TABLES/5. PMC3610192 23497695

[6] Katz AJ, Santoro M, Diblasio F, Ashley R. Stereotactic body radiotherapy for localized prostate cancer: disease control and quality of life at 6 years. 2013. 10.1186/1748-717X-8-118. PMC3674983 23668632

[7] Byrne M, et al. Intrafraction motion and margin assessment for ethos online adaptive radiotherapy treatments of the prostate and seminal vesicles. Adv Radiat Oncol. Mar. 2024;9(3):101405. 10.1016/J.ADRO.2023.101405. PMC10831186 38304111

[8] Sihono DSK, et al. Determination of intrafraction prostate motion during external beam radiation therapy with a transperineal 4-dimensional ultrasound real-time tracking system. Int J Radiat Oncol Biol Phys. 2018;101(1):136–43. 10.1016/j.ijrobp.2018.01.040. 29482869

[9] Shimomura A, et al. Monitoring intrafraction motion of the prostate during radiation therapy: suggested practice points from a focused review. Pract Radiat Oncol. Mar. 2024;14(2):146–53. 10.1016/J.PRRO.2023.08.017/ATTACHMENT/CB845B7B-6D4B-44FB-9437-6BA9B7B58226/MMC1.DOCX. 37875222

[10] Nicosia L, Alongi F, Rigo M, Cuccia F, Giaj-Levra N, Mazzola R, et al. Prostate volume variation during 1.5T MR-guided adaptive stereotactic body radiotherapy (SBRT) and correlation with treatment toxicity. Radiother Oncol. 2024 Jan;190:110009 10.1016/j.radonc.2023.110043. 38056694

[11] De Roover R, et al. Automated treatment planning of prostate stereotactic body radiotherapy with focal boosting on a fast-rotating O-ring linac: Plan quality comparison with C-arm linacs. J Appl Clin Med Phys. 2021;22(9):59–72. 10.1002/acm2.13345. PMC8425873 34318996

[12] Byrne M, et al. Varian ethos online adaptive radiotherapy for prostate cancer: Early results of contouring accuracy, treatment plan quality, and treatment time. J Appl Clin Med Phys. 2022;23(1). 10.1002/acm2.13479. PMC8803282 34846098

[13] Waters M, Patel R, Wilkins A, Tree AC, McNair HA, Hansen VN, et al. CT-based online adaptive radiotherapy improves target coverage and organ at risk (OAR) avoidance in stereotactic body radiation therapy (SBRT) for prostate cancer. Clin Transl Radiat Oncol. 2024 Jan;44:100693. 10.1016/j.ctro.2023.100693. PMC10663731 38021093

[14] Fischer J, et al. CBCT-based online adaptive radiotherapy of the prostate bed: first clinical experience and comparison to nonadaptive conventional IGRT. Strahlentherapie und Onkol. 2024. 10.1007/s00066-024-02323-6. PMC12283463 39499306

[15] Moazzezi M, Rose B, Kisling K, Moore KL, Ray X. Prospects for daily online adaptive radiotherapy via ethos for prostate cancer patients without nodal involvement using unedited CBCT auto-segmentation. J Appl Clin Med Phys. 2021;22(10):82–93. 10.1002/acm2.13399. PMC8504605 34432932

[16] Zwart LGM, et al. Cone-beam computed tomography-guided online adaptive radiotherapy is feasible for prostate cancer patients. Phys Imaging Radiat Oncol. 2022;22:98–103. 10.1016/j.phro.2022.04.009. PMC9115122 35602545

[17] Gan G, et al. A study of criteria-based online adaptive radiotherapy with radiomics and dosimetry for postoperative prostate cancer. Med Phys. Aug. 2025;52(8). 10.1002/mp.18058. PMC12356990 40817276

[18] Malygina H, Salazar Zuniga B, Auerbach H, Ries M, Dzierma Y, Hecht M, et al. Daily online adaptation enhances target coverage in prostate cancer radiotherapy: a retrospective analysis. Front Oncol. 2025;15:1662671. 10.3389/fonc.2025.1662671. PMC12620223 41256324

[19] Consortium SABRUK. Stereotactic ablative body radiation therapy (SABR): A Resource Version 5.0. 2015;1–99 [Online]. Available: https://www.actionradiotherapy.org/wp-content/uploads/2014/12/UKSABRConsortiumGuidellinesv5.pdf.

[20] Jiang D, Wang Y, Zhang Y, Li X, Liu H, Chen J, et al. First implementation and results of online adaptive radiotherapy for cervical cancer based on CT-Linac combination. Front Oncol. 2024;14:1475468. 10.3389/fonc.2024.1399468. PMC11602513 39610921

[21] Åström LM, et al. Online adaptive radiotherapy of urinary bladder cancer with full re-optimization to the anatomy of the day: Initial experience and dosimetric benefits: oART of bladder cancer: initial experience and dosimetric benefits. Radiother Oncol. 2022;171:37–42. 10.1016/j.radonc.2022.03.014. 35358605

[22] Blumenfeld P, et al. Real world clinical experience using daily intelligence-assisted online adaptive radiotherapy for head and neck cancer. Radiat Oncol. 2024;19(1):1–7. 10.1186/s13014-024-02436-3. PMC10981810 38555453

[23] Malygina H, et al. Intra-adaptational changes in online adaptive radiotherapy: from the ideal to the real dose. Strahlentherapie und Onkol. 2025. 10.1007/s00066-025-02425-9. PMC12546291 40663147

[24] Fischer J, et al. CBCT-based online adaptive radiotherapy of the bladder – geometrical and dosimetrical considerations compared to conventional IGRT. Radiat Oncol. Dec. 2025;20(1):1–13. 10.1186/S13014-025-02710-Y/FIGURES/10. PMC12351964 40813674

[25] Preziosi F, et al. AI-driven online adaptive radiotherapy in prostate cancer treatment: considerations on activity time and dosimetric benefits. Radiat Oncol. 2025;20(1). 10.1186/s13014-025-02697-6. PMC12296583 40713724

[26] Brand DH, et al. Intensity-modulated radiotherapy versus stereotactic body radiotherapy for prostate cancer (PACE-B): 2-year toxicity results from an open-label, randomised, phase 3, non-inferiority trial. Lancet Oncol. 2022;23:1308–20. 10.1016/S1470-2045(22)00517-4. 36113498

[27] Alongi F, et al. 1.5 T MR-guided and daily adapted SBRT for prostate cancer: Feasibility, preliminary clinical tolerability, quality of life and patient-reported outcomes during treatment. Radiat Oncol. Mar. 2020;15(1):1–9. 10.1186/S13014-020-01510-W/TABLES/7. PMC7092497 32248826

[28] di Franco F, et al. Ultra-hypofractionated prostate cancer radiotherapy: Dosimetric impact of real-time intrafraction prostate motion and daily anatomical changes. Phys Med. Feb. 2024;118:103207. 10.1016/j.ejmp.2024.103207. 38215607

