# Supplementary material for: CBCT based online daily adaptive treatment improves target coverage with limited benefit for OAR protection in SBRT prostate cancer
Source: Radiat Oncol. 2026 Jul 7;21:130. doi: 10.1186/s13014-026-02888-9 (PMC13629108; doi:10.1186/s13014-026-02888-9)
Supplement: Supplementary file 1 [file 13014_2026_2888_MOESM1_ESM.docx]

| **Evaluated Volumens** | **Dose Constrains** |
| --- | --- |
| CTVpros_V100%_ | ≥ 95% |
| CTVpvs_V100%_ | ≥ 95% |
| PTVpros_V100%_ | ≥ 90% |
| PTVpros_V95%_ | ≥ 99% |
| PTVpros_D0.01_ | ≥ 99% |
| PTVpros_Dmean_ | ≥ 110% |
| PTVpvs_V100%_ | ≥ 90% |
| PTVpvs_V95%_ | ≥ 99% |
| Bladder wall_D15cm3_ | < 18.3Gy |
| Bladder wall_D0.04 cm3_ | < 38Gy |
| Rectum_D1cm3_ | < 36Gy |
| Rectum_D0.04cm3_ | < 38Gy |
| Rectum_D50%_ | < 18.1Gy |
| Rectum_D20%_ | < 29Gy |
| Bladder_D40%_ | < 18.1Gy |
| Bladder_V37Gy_ | < 5cm^3^ |
| Penile bulb_D5cm3_ | < 30Gy |
| Penile bulb_D3cm3_ | < 30Gy |
| Sigmoid colon_D0.04cm3_ | < 38Gy |
| Sigmoid colon_D20cm3_ | < 25Gy |
| Femur h. Left_D10cm3_ | < 30Gy |
| Femur h. Right_D10cm3_ | < 30Gy |
